# A case report of a retained interventricular septal bullet after gunshot wound

**DOI:** 10.1093/jscr/rjab179

**Published:** 2021-05-17

**Authors:** Brandon M Smith, Elisabeth Loomis, Logan Erz, Ted F Shaub, Eric Espinal, Rathna Shenoy

**Affiliations:** Department of Trauma, Akron City Hospital, Akron, OH, USA; Department of Trauma, Akron City Hospital, Akron, OH, USA; Department of Trauma, Akron City Hospital, Akron, OH, USA; Department of Cardiology, Akron City Hospital, Akron, OH, USA; Department of Cardiothoracic Surgery, Akron City Hospital, Akron, OH, USA; Department of Trauma, Akron City Hospital, Akron, OH, USA

## Abstract

Cardiac gunshot injuries herald a universally grim prognosis. We present an exceedingly unique case of a patient surviving multiple gunshot wounds with two bullet fragments lodged in the interventricular septum.

A 25-year-old male sustained four gunshot wound injuries to the upper body. Two cardiac interventricular septal bullet fragments were identified during his recovery. Management included serial echocardiographic surveillance and a two-month regimen of empiric colchicine for prophylaxis against post-traumatic pericarditis.

Pursuing non-operative management especially in asymptomatic or stable patients should be evaluated against surgical extraction and possible sequelae of complications. The consideration of scheduled colchicine for pericarditis prophylaxis is warranted as well as interval echocardiogram.

Retained myocardial bullets are exceedingly rare clinical events with scant literature available to guide clinical decisions. Management requires intricate decision-making and close consideration of risk benefit analysis weighing surgical extraction against non-operative management.

## INTRODUCTION

Management of penetrating thoracic trauma requires team-based, high-quality medical care. These injuries account for 1–13% of all trauma admissions with 5–15% requiring emergent thoracic exploration [1]. Cardiac gunshot injuries necessitating resuscitative thoracotomy carry a near 100% universal mortality rate [2]. Patients stable enough for non-resuscitative thoracotomy (in the operating room) have an improved overall survival rate approaching 62% [2].

Given this clinical scenario, scant literature exists to guide management decisions and is limited to a handful of case reports describing retained bullet fragments scattered throughout various locations in the heart [3–6]. We describe an unique case of a patient surviving multiple upper body gunshot wounds including two bullet fragments lodged in the interventricular septum and discuss the management considerations of retained intracardiac bullets including the use of colchicine for pericarditis prophylaxis.

## CASE PRESENTATION

**Figure 1:**
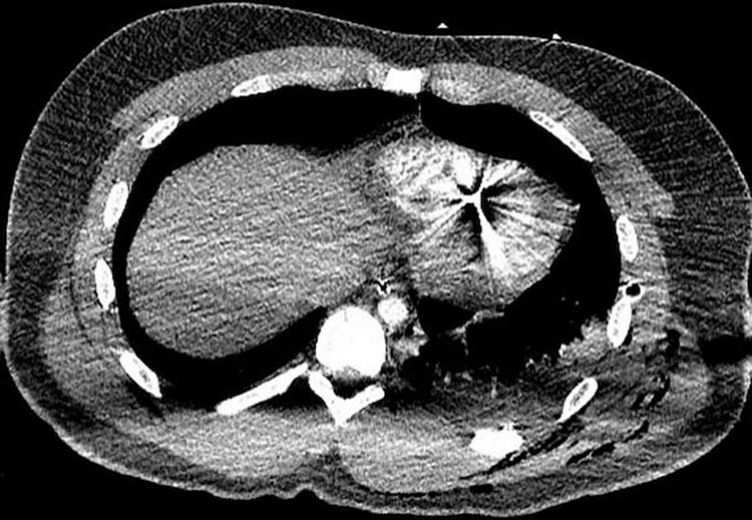
Axial CT scan of the chest identifying bullet fragment in the interventricular septum.

**Figure 2:**
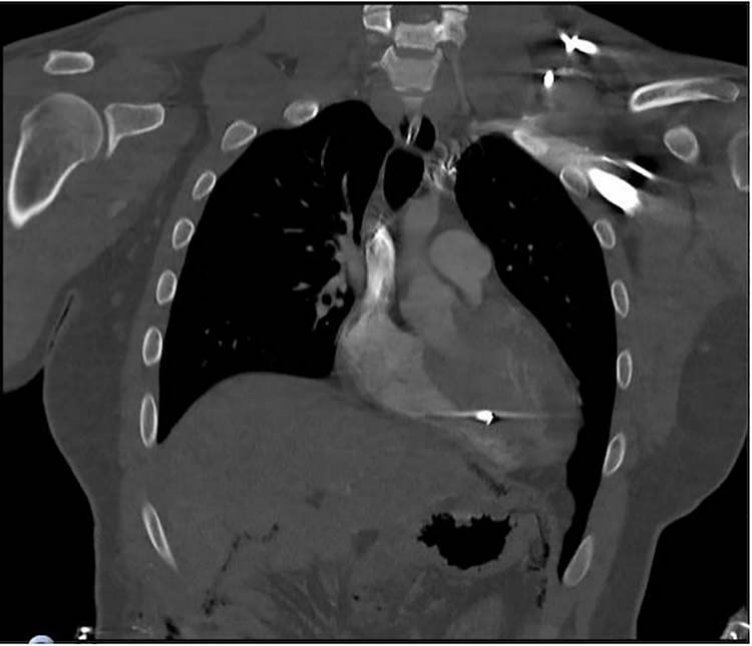
Coronal CT scan of the chest identifying bullet fragment in the interventricular septum.

A 25-year-old male presented to the emergency department with four gunshot wounds to the right hip, right flank, left flank and left posterior thorax. Upon arrival the patient was hemodynamically stable with a Glasgow coma score of 15. Chest and abdominal plain films demonstrated a left-sided hemothorax, scattered bullet fragments within the left hemithorax and multiple bullet fragments in the abdominal cavity. A left tube thoracostomy was placed yielding immediate output of 575cc sanguineous fluid and the patient was taken for an exploratory laparotomy. A left diaphragmatic defect was identified with splenic herniation into the left thorax. Splenectomy and primary diaphragmatic repair were performed. Multiple bowel injuries were encountered necessitating several segmental resections. The next day a computed tomography (CT) scan of the chest, abdomen and pelvis was obtained, which identified presence of two bullet fragments in the interventricular septum (Figs 1 and 2).

**Figure 3:**
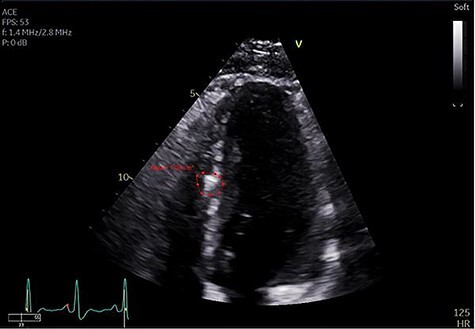
Echocardiogram identifying bullet fragment in the interventricular septum.

The patient recovered without complications other than a persistent, asymptomatic, sinus tachycardia with heart rate sustained at 120 beats per minute. On post-trauma Day 10 an echocardiogram was obtained, which again demonstrated two bullet fragments retained within the interventricular septum, a small pericardial effusion without evidence of tamponade and no evidence of ventricular septal defect (Fig. 3). Non-operative management of the retained septal projectiles was pursued. Empiric colchicine 0.6 mg twice daily for post-traumatic pericarditis prophylaxis was prescribed. Four days later the patient’s tachycardia resolved spontaneously. Point of care ultrasound demonstrated resolution of the pericardial effusion. The patient was discharged from the hospital on post-trauma Day 19 with a 2-month course of colchicine. At outpatient follow-up, he continued to maintain normal sinus rhythm without evidence of effusion or pericarditis.

## DISCUSSION

Penetrating cardiac gunshot injuries yield an extremely high mortality rate approaching 100% for patients requiring emergency department thoracotomy [2]. Select survivors of this traumatic injury require complex critical care, and the presence of post-traumatic-retained intracardiac bullets provides an additional management dilemma.

Although multiple factors dictate patient management, the root decision surrounding retained intracardiac foreign bodies is surgical extraction versus observation (expectant management). The presence of a retained intracardiac foreign body carries the potential for the development of complications. Immediate potential sequelae include myocardial infarction, cardiogenic shock, pericardial tamponade, and extracardiac bullet embolism [3, 6]. Post-traumatic myocardial infarction, cardiogenic shock, and pericardial tamponade are immediate life-threatening emergencies requiring emergent intervention. Bullet embolization is a much less common clinical event with incidence of 0.3% after injuries involving the arterial system and carries additional morbidity [7]. When pursuing extraction, the common surgical approach for thoracic exploration is median sternotomy with cardiopulmonary bypass and ventriculotomy [5] or atriotomy [3] as dictated by the bullet location. Fragomeni and Azambuja [6] reported three cardiac bullet extractions, all performed on cardiopulmonary bypass via cannulation of the aorta, superior and inferior vena cava, aortic cross-clamp and cardioplegia solution infusion. This extensive surgical approach adds to morbidity; therefore, in stable patients expectant management may be appealing. Long-term complications of retained intracardiac projectiles include valvular dysfunction, septal defects, effusion, pericarditis or bullet migration. Although there is no standardized surveillance protocol, close follow-up and interval echocardiograms have been described to monitor for these long-term complications [3].

Duong *et al* reported a patient with a retained bullet in the right ventricle 37 years after sustaining a mid-back gunshot wound. Their patient presented with dyspnea and chest pain and was found to have a bullet retained in the right ventricle. The bullet was in a stable, unchanging location without arrhythmias or tamponade and the patient was managed with observation [4]. Our patient’s cardiac injury included two retained bullet fragments in the interventricular septum, a small pericardial effusion without tamponade, and spontaneous resolution of sinus tachycardia. The fact that the bullets were not initially identified at the index operation further supported the decision to continue with observation, which includes serial echocardiograms to assess for development of long-term complications including valvular dysfunction, delayed septal defects and bullet migration.

We added empiric colchicine therapy for pericarditis prophylaxis. Post-traumatic pericarditis is usually self-limiting but occasionally can cause constrictive pericarditis or cardiac tamponade [6, 8]. The use of colchicine in treating acute pericarditis and preventing recurrent pericarditis or post-pericardiotomy syndrome has been well described in the literature [9, 10]; however, there is little information advocating for or against the use of colchicine to reduce post-traumatic pericarditis. We empirically treated our patient with colchicine for 2 months and achieved resolution of his tachycardia and pericardial effusion after starting treatment.

## CONCLUSION

This case of retained interventricular septum myocardial bullet fragments after gunshot injury adds to the literature describing a unique clinical challenge that trauma surgeons may face. Retained myocardial bullets are extremely uncommon clinical findings with little literature available to guide management decisions. As demonstrated in this case, the management pathway involves a risk–benefit analysis and, in select cases, non-operative management. This case serves as an example of successful non-operative management with the concurrent use of empiric colchicine for post-traumatic pericarditis prophylaxis. Stable patients with retained intracardiac bullets can be considered for non-surgical treatment with observation if close follow-up and surveillance with echocardiograms are arranged. Additionally, the consideration of scheduled colchicine may be beneficial in preventing post-traumatic pericarditis, although further research is warranted to qualify this potential benefit.

## CONFLICT OF INTEREST STATEMENT

None declared.
